# Analysis of intraoperative modifiable factors to prevent acute kidney injury after elective noncardiac surgery: intraoperative hypotension and crystalloid administration related to acute kidney injury

**DOI:** 10.1186/s40981-021-00429-9

**Published:** 2021-03-24

**Authors:** Yasuma Kobayashi, Kazue Yamaoka

**Affiliations:** 1 Department of Anesthesiology and Critical Care Medicine, Jichi Medical University Saitama Medical Center, 1-847 Amanumacho, Omiya-ku, Saitama, Saitama, 330-8503 Japan; 2 Department of Anesthesiology, Saitama Children’s Medical Center, 1-2, Shintoshin, Chuo-ku, Saitama, Saitama, 3308777 Japan; 3 Teikyo University Graduate School of Public Health, 2-11-1 Kaga Itabashi-ku, Tokyo, 173-8605 Japan

**Keywords:** Intraoperative hypotension, Crystalloid administration, Acute kidney injury

## Abstract

**Background:**

The optimal intraoperative blood pressure range and crystalloid administration protocol for the prevention of acute kidney injury (AKI) after elective noncardiac surgery remain unknown.

**Methods:**

This single-center retrospective cohort study included 6296 patients aged ≥ 50 years who had undergone elective noncardiac surgery under general anesthesia. We evaluated the relationship between duration of intraoperative hypotension and AKI. To assess whether the effects of crystalloid administration differed according to baseline estimated glomerular filtration rate (eGFR), we examined the interaction between intraoperative crystalloid administration and eGFR. We calculated univariable and multivariable adjusted odds ratios (ORs) and their 95% confidence intervals (95% CIs) for the prevalence of AKI.

**Results:**

AKI occurred in 431 (6.8%) patients and was associated with intraoperative hypotension. Effects of intraoperative crystalloid administration differed significantly according to baseline eGFR. Increased risk of AKI was noted in patients with eGFR ≤45 ml min^−1^ 1.73m^−2^ who were managed with restrictive or liberal crystalloid administration [OR 4.79 (95% CI 3.10 to 7.32) and 6.43 (95% CI 2.23 to 16.03), respectively] as opposed to those with eGFR >45 ml min^−1^ 1.73m^−2^ who were managed with moderately restrictive crystalloid administration.

**Conclusions:**

Our findings suggest that anesthesiologists should avoid intraoperative hypotension as well as either restrictive or liberal (as opposed to moderately restrictive) crystalloid administration in patients with decreased eGFR. Intraoperative blood pressure and crystalloid administration protocol are major modifiable factors that must be optimized to prevent postoperative AKI.

**Supplementary Information:**

The online version contains supplementary material available at 10.1186/s40981-021-00429-9.

## Introduction

Postoperative acute kidney injury (AKI) has a detrimental effect on patient outcome: it has been associated with higher risk of chronic kidney disease (CKD) [1–3] and with higher likelihood of both early and long-term mortality [4]. Yet AKI remains one of the more common organ injuries after surgery, with a reported incidence in Turkey of 6.7% after noncardiac surgery [5]. Appropriate intraoperative blood pressure management and crystalloid administration practices are major modifiable factors that anesthesiologists can adjust to prevent postoperative AKI and maintain organ perfusion. To date, however, the results regarding optimal intraoperative blood pressure and crystalloid administration protocols have been contradictory.

Appropriate management of intraoperative blood pressure is an important part of preventing postoperative AKI [6–9]. A systematic review has shown that interventions aimed at avoiding intraoperative hypotension (IOH) reduced the incidence of postoperative AKI [10]. Another study assessing definitions of IOH based on absolute and relative thresholds showed that associations based on relative mean arterial pressure thresholds were no stronger than those based on absolute thresholds [8]. A recent study has shown that systolic blood pressure below a threshold of roughly 90 mmHg was associated with postoperative AKI [11]. To date, there have been no studies investigating the relationship between IOH and postoperative AKI in Japan, however.

Identifying an optimal approach to crystalloid administration is another important step in preventing postoperative AKI [12]. A meta-analysis has shown that interventions aimed at optimizing perioperative hemodynamic status reduced the incidence of postoperative AKI [13], while an RCT has shown that restrictive crystalloid administration increased the incidence of postoperative AKI [14]. However, these previous studies have not assessed whether the effects of crystalloid administration differ according to baseline renal function.

The aims of the present study were (i) to investigate the effects of IOH (defined based on either an absolute or a relative threshold) on AKI after elective non-cardiac surgery and (ii) to investigate whether the effects of crystalloid administration differ according to baseline renal function. As far as we know, this is the first study to examine the effects of IOH and intraoperative crystalloid administration on AKI in Japan.

## Methods

### Ethical approval

This study was approved by the institutional review boards (IRB) of Jichi Medical University Saitama Medical Center, Saitama, Japan, (approval no S18-042; Chairperson: Prof. R. Nagai; approval date: 2 August 2018) and Teikyo University, Tokyo, Japan, (approval no. 18-106; Chairperson: Prof. M. Kawamura; approval date: 25 September 2018), and the requirement for written informed consent was waived by the IRBs.

### Study design

We conducted a retrospective cohort study in compliance with the applicable Strengthening the Reporting of Observational Studies in Epidemiology statement. We screened data on patients who had undergone elective noncardiac surgery under general anesthesia at Jichi Medical University Saitama Medical Center, a 628-bed tertiary care center in Saitama, Japan, that performs around 6500 operations a year, and who satisfied our selection criteria. Data from April 1, 2009, through March 31, 2018, were used in this study.

### Study population

We included patients who met all of the following criteria: (1) patients aged ≥ 50 years who had undergone elective noncardiac surgery under general anesthesia between April 1, 2009, and March 31, 2018; (2) duration of anesthesia was >60 min. We limited our study to patients aged ≥ 50 years because elderly patients are at higher risk of postoperative AKI. We excluded patients who met one of the following criteria: (1) urological surgery; (2) for patients with multiple surgeries during the study period, we included the first surgery only; (3) missing baseline or postoperative creatinine values; (4) eGFR <15 ml min^−1^ 1.73m^−2^; (5) American Society of Anesthesiologists Physical Status (ASAPS)=1.

### Data sources

The data used in this study were extracted from two databases, namely, the electronic medical systems software Cosmos (COSMOS, IBM Japan, Ltd., Japan) and the anesthesia record system ORSYS (Philips Electronics Japan, Ltd., Japan). Preoperative creatinine, postoperative creatinine, preoperative systolic blood pressure (SBP), and diastolic blood pressure (DBP) were extracted from Cosmos using SQL language. Preoperative blood pressure was measured with the patient at rest in the ward the day before surgery with a Terumo electronic sphygmomanometer ES-H55 [15]. Intraoperative blood pressure data and patient demographic information were extracted from ORSYS using the Vipros data-mining software (DOWELL Co., Ltd., Japan) [16]. Intraoperative blood pressure values were measured with a Philips IntelliVue MP70.

### Primary outcome

The primary endpoint was the development of postoperative AKI. Based on the definition proposed in the Kidney Disease: Improving Global Outcome guidelines, AKI was defined as the presence of one or both of the following: (i) increase in serum creatinine by ≥0.3 mg dl^−1^ within 48 h and/or (ii) increase in serum creatinine to ≥1.5 times baseline within 7 days [17].

### Potential confounding factors

As potential confounding factors, we identified variables related to patients’ baseline characteristics, coexisting medical conditions, and intraoperative factors. Baseline patient characteristics included sex, age, body mass index (BMI), body weight, ASAPS, and cardiac medication histories. Cardiac medication histories included the use of angiotensin-converting enzyme inhibitors (ACE inhibitors), angiotensin receptor blockers (ARBs), beta blockers, calcium channel blockers (CCBs), and diuretics. We also collected data on preoperative SBP and eGFR as defined by Cockcroft-Gault and adjusted for body surface area by normalizing the output per 1.73 m^2^ of body surface area. Coexisting medical conditions included information on congestive heart failure, hypertension, coronary artery disease, valvular heart disease, diabetes mellitus, dyslipidemia, cerebrovascular disease, spinal cord injury, chronic obstructive pulmonary disease, interstitial pneumonia, mixed obstructive and restrictive lung disease, respiratory failure, cirrhosis, coagulopathy, hyperthyroidism, neuromuscular diseases, Parkinson’s disease, depression, schizophrenia, dementia, and ileus. Intraoperative factors included anesthesia time, hemorrhage, crystalloid administration (ml kg^−1^ h^−1^), urine (as a categorical value, ml kg^−1^ h^−1^), type of surgical procedure, and the use or non-use of combined epidural anesthesia, invasive arterial catheter, 0.9% saline infusion, synthetic colloid infusion, and transfusion of red blood cells (RBC) and/or fresh frozen plasma (FFP). In previous observational studies investigating the effects of IOH, the volume of intraoperative crystalloid administration was not adjusted for as a confounding factor. However, a recent RCT showed that restrictive crystalloid administration was associated with postoperative AKI [18]. Administration of 0.9% saline is traditionally considered to increase the risk of postoperative AKI [19]. In this study, “crystalloid” refers to buffered crystalloid, and 0.9% saline and buffered crystalloids were analyzed separately.

As for the type of surgical procedure, the data originally identified 535 distinct procedure names. We combined them into the following 12 broader categories: major abdominal surgery, laparoscopic major abdominal surgery, gynecological surgery, thoracic surgery, major vascular surgery, peripheral vascular surgery, neurosurgery, spinal surgery, orthopedic surgery, head and neck surgery, other high-risk surgeries, and other low-risk surgeries [18, 20]. The surgical procedures included in the other high-risk surgery and other low-risk surgery categories are listed in Table 1S (Supplemental Digital Content 1).

### Statistical analyses

Patients’ baseline characteristics, coexisting medical conditions, intraoperative factors, and surgical procedure types were summarized as means and standard deviations (SD) for continuous variables and as frequencies and proportions for nominal variables, according to the presence or absence of postoperative AKI.

Our analysis consisted of two steps. In step 1, we prepared the data and evaluated the effects of duration of IOH (defined according to absolute or relative threshold) on AKI using univariable and multivariable logistic regression models. In step 2, we investigated whether the effects of crystalloid administration differed according to baseline renal function. The details of each step are presented below.

#### Step 1: removing artifacts and examining the association between IOH and postoperative AKI

Blood pressure was measured non-invasively at 1- to 5-min intervals and invasively at 1-min intervals. When both non-invasive and invasive blood pressure measurements were available for the same time point, we used invasive measurements. We removed out-of-range blood pressure data as artifactual according to the following exclusion criteria [6].

1. SBP ≥300 mmHg or ≤35 mmHg
2. DBP ≥225 mmHg or ≤5 mmHg
3. Difference between SBP and DBP <10 mmHg
4. SBP rises or falls by ≥110 mmHg within 1 min

After artifact removal, blood pressure values between measurements were linearly interpolated according to 1-min interval data.

We examined the relationship between IOH and postoperative AKI using univariable and multivariable logistic regression models for both absolute and relative thresholds. We tested five different absolute thresholds of SBP: 70 mmHg, 75 mmHg, 80 mmHg, 85 mmHg, and 90 mmHg. The ORs and the 95% CIs of postoperative AKI were calculated according to the logistic regression model for each of four duration categories: 0 min (reference), 1 to 5 min, 5 to 20 min, and >20 min. We chose these four categories in keeping with previous studies to assess the effects of both short and long periods of IOH. Modeling duration as a continuous variable, we tested for trends in how the OR increased. As relative SBP thresholds, we tested 65%, 70%, 75%, 80%, and 85% of baseline. The same analysis used to assess the absolute values was also conducted for these relative thresholds. The covariates included in the final model were determined by a stepwise selection method with inclusion and exclusion criteria of 0.2. All of the covariates included in step 1 were also included in step 2.

#### Step 2: examining whether the effects of crystalloid administration differ according to baseline renal function

We determined whether the effects of crystalloid administration differed according to baseline renal function by including an interaction term between crystalloid administration and baseline renal function into the model used in step 2. In this model, we used an absolute threshold for blood pressure. We classified baseline renal function into two categories: eGFR <45 ml min^−1^ 1.73m^−2^ (reference is 45 ml min^−1^ 1.73m^−2^≤ eGFR) and eGFR <60 ml min^−1^ 1.73m^−2^ (reference is 60 ml min^−1^ 1.73m^−2^≤ eGFR) according to K/DOQI practice guidelines [21]. We did not include patients with eGFR <30 ml min^−1^ 1.73m^−2^ in this model because the sample size for this population was too small. We also classified patients into three categories according to the volume of crystalloid administration: liberal (>24 ml kg^−1^ h^−1^), moderately restrictive (>7 ml kg^−1^ h^−1^, ≤24 ml kg^−1^ h^−1^, reference), and restrictive (≤7 ml kg^−1^ h^−1^) [22].

#### Sensitivity analysis

As a sensitivity analysis, we conducted the same analyses described in step 1 using mean arterial pressure (MAP) instead of SBP.

#### Sample size considerations

We assumed a postoperative AKI incidence of 5% and defined a difference of 2–5% associated with the presence or absence of IOH as clinically important. With a two-sided α error of 0.05 and a power of 0.8, 435 to 2213 patients were required for each arm of the study. A two-tailed *p*-value of 0.05 was considered to indicate statistical significance in all analyses. Statistical analyses were performed using SAS Ver. 9.4.

## Results

### Characteristics of study population

**Fig. 1:**
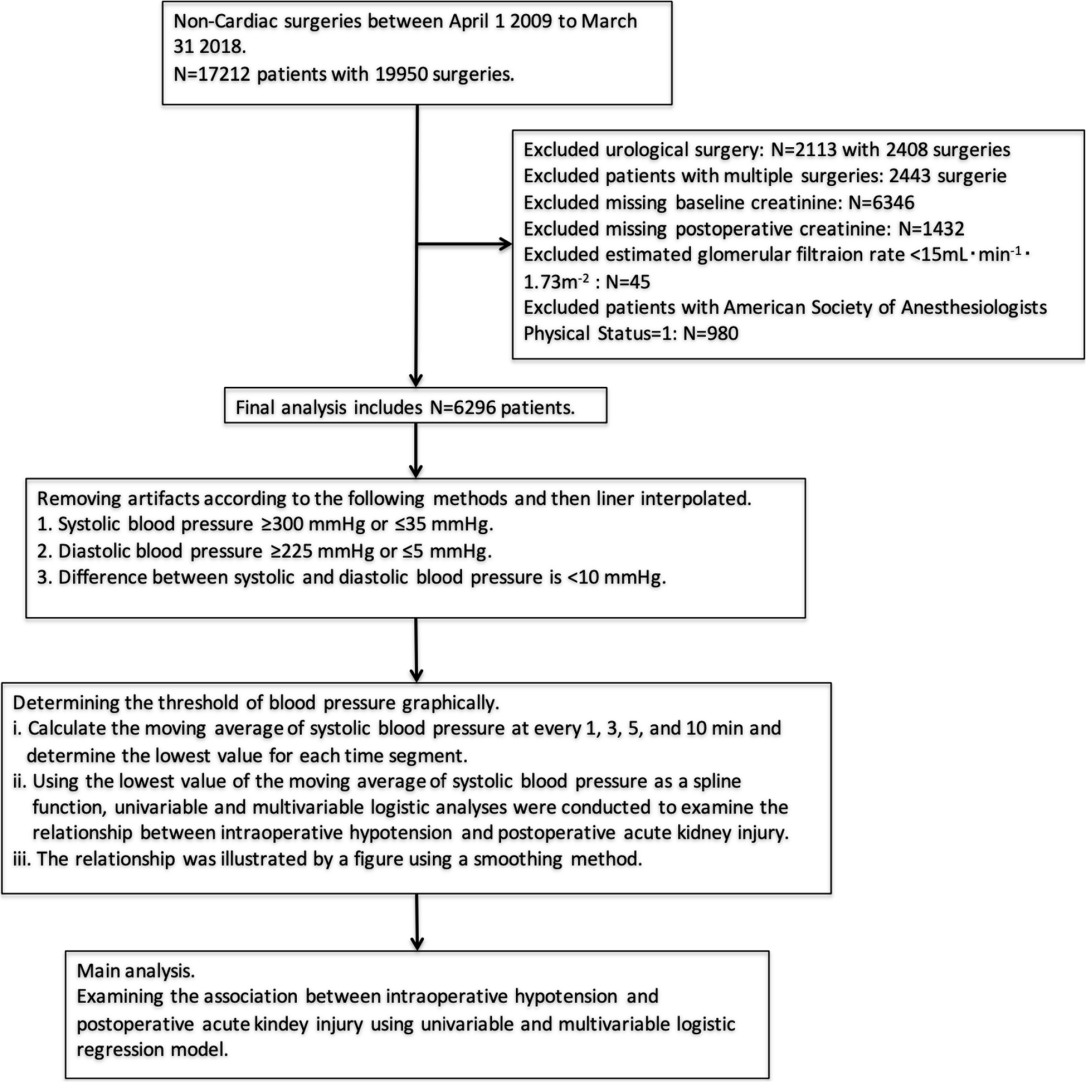
Flow chart of study procedure

Figure 1 is a flow chart showing the study procedures. Of the 6296 patients included in the final analysis, postoperative AKI occurred in 431 (6.8%) patients. Table 1 is a summary of the patients’ baseline characteristics, coexisting medical conditions, and intraoperative factors. Patients who developed postoperative AKI were more likely to be male and to use cardiac medications, and to have higher ASAPS scores, lower eGFR, congestive heart failure, hypertension, coronary artery disease, valvular heart disease, diabetes mellitus, cerebrovascular disease, cirrhosis, and/or depression (*p*<0.05 in all cases). As for intraoperative factors, patients with postoperative AKI were likely to have longer anesthesia time and greater hemorrhage volume, and were more likely to receive 0.9% saline administration, synthetic colloid administration, RBC and FFP transfusion, combined epidural anesthesia, and arterial catheter (*p*<0.05 in all cases). As for surgical procedures, patients with postoperative AKI were more likely to have undergone major abdominal surgery, major vascular surgery, or other high-risk surgeries, and less likely to have undergone laparoscopic major abdominal surgery, thoracic surgery, neurosurgery, spinal surgery, and other low-risk surgeries.

**Table 1:** Patients’ baseline characteristics, coexisting medical conditions, intraoperative characteristics, and surgical procedures by acute kidney injury (*N*=6296)

		Non-AKI (*n*=5865)	AKI (*n*=431)	*p* value
**Baseline patient characteristics**				
Sex (female), *n* (%)		2143 (36.54)	107 (24.83)	< 0.0001
Age, years	≥50, <65	1515 (25.83)	90 (20.88)	0.0840
	≥65	4350 (74.17)	341 (79.12)	0.4113
BMI, kg m^−2^	<25	1621 (27.64)	127 (29.47)	0.5769
	≥25	4244 (72.36)	304 (70.53)	0.7709
ASAPS, *n* (%)	2	4955 (84.50)	326 (75.60)	0.1555
	3	868 (14.80)	98 (22.70)	0.0003
	4	42 (0.72)	7 (1.62)	0.0778
Cardiac medication histories, *n* (%)	ACE inhibitors	393 (6.70)	51 (11.83)	<0.0001
	ARBs	1651 (28.15)	148 (34.34)	0.0072
	Beta blockers	878 (14.97)	115 (26.68)	<0.0001
	CCBs	2177 (37.10)	211 (48.90)	<0.0001
	Diuretics	481 (8.20)	73 (16.94)	<0.0001
Baseline systolic BP, mmHg		123 ± 17.8	125 ±19.6	0.2300^*a*^
Baseline eGFR, ml min^−1^ 1.73m^−2^	15≤ <45	623 (10.62)	116 (26.91)	<0.0001
	≥45	5242 (89.38)	315 (73.09)	0.0094
**Coexisting medical conditions**				
Congestive heart failure, *n* (%)		330 (5.63)	35 (8.12)	0.0422
Hypertension, *n* (%)		3275 (55.84)	298 (69.14)	<0.0001
Coronary artery disease, *n* (%)		607 (10.35)	62 (14.39)	0.0110
Valvular heart disease, *n* (%)		401 (6.84)	42 (9.74)	0.0292
Diabetes mellitus, *n* (%)		1392 (23.73)	127 (29.47)	0.0086
Dyslipidemia, *n* (%)		1550 (26.43)	120 (27.84)	0.5583
Cerebrovascular disease, *n* (%)		472 (8.05)	52 (12.06)	0.0047
Spinal cord injury, *n* (%)		6 (0.10)	1 (0.23)	0.9751
COPD, *n* (%)		945 (16.11)	57 (13.23)	0.1302
Interstitial pneumonia, *n* (%)		397 (6.77)	28 (6.50)	0.9060
Mixed obstructive and restrictive lung disease, *n* (%)		90 (1.53)	11 (2.55)	0.1543
Respiratory failure, *n* (%)		354 (6.04)	16 (3.71)	0.0610
Cirrhosis, *n* (%)		53 (0.90)	11 (2.55)	0.0023
Coagulopathy, *n* (%)		159 (2.71)	17 (3.94)	0.1777
Hyperthyroidism, *n* (%)		114 (1.94)	10 (2.32)	0.7164
Neuromuscular disease, *n* (%)		32 (0.55)	1 (0.23)	0.5998
Parkinson’s disease, *n* (%)		18 (0.31)	3 (0.70)	0.3578
Depression, *n* (%)		55 (0.94)	11 (2.55)	0.0034
Schizophrenia, *n* (%)		19 (0.32)	1 (0.23)	1.0000
Dementia, *n* (%)		62 (1.06)	3 (0.70)	0.6392
Ileus, *n* (%)		15 (0.26)	3 (0.70)	0.2360
**Intraoperative factors**				
Anesthesia time, min	>60, ≤278	1480 (25.23)	46 (10.67)	<0.0001
	>278, ≤372	1499 (25.56)	53 (12.30)	<0.0001
	>372, ≤501	1486 (25.34)	125 (29.00)	0.2205
	501<	1400 (23.87)	207 (48.03)	<0.0001
Hemorrhage, ml	0	1457 (24.84)	44 (10.21)	<0.0001
	>0, ≤100	1283 (21.88)	37 (8.58)	<0.0001
	>100, ≤330	1739 (29.65)	107 (24.83)	0.1229
	>330	1386 (23.63)	243 (56.83)	<0.0001
Crystalloid, ml kg^−1^ h^−1^	≥7, <24	3950 (67.34)	263 (61.02)	0.2380
	≥24	168 (2.86)	11 (2.55)	0.8280
	≥0, <7	1747 (29.78)	157 (36.42)	0.0420
0.9% saline, *n* (%)		2383 (40.63)	223 (51.74)	<0.0001
Synthetic colloid, *n* (%)		2094 (35.70)	235 (54.52)	<0.0001
Transfusion, *n* (%)	RBC	486 (8.29)	115 (26.68)	<0.0001
	FFP	140 (2.39)	43 (9.98)	<0.0001
Urine, ml kg^−1^ h^−1^	≥0.5	4475 (76.30)	314 (72.85)	0.1188
	≥0, <0.5	1390 (23.70)	117 (27.15)	0.2314
Combined epidural anesthesia, *n* (%)		2371 (40.43)	196 (45.48)	0.0446
Use of arterial catheter, *n* (%)		4280 (73.10)	361 (83.76)	<0.0001
**Surgical procedures**				
Major abdominal		1263 (21.53)	190 (44.08)	<0.0001
Laparoscopic major abdominal		833 (14.20)	35 (8.12)	0.0021
Gynecological		160 (2.73)	6 (1.39)	0.1389
Thoracic		1566 (26.70)	42 (9.74)	<0.0001
Major vascular		617 (10.52)	94 (21.81)	<0.0001
Peripheral vascular		205 (3.50)	12 (2.78)	0.5354
Neurosurgery		381 (6.50)	9 (2.09)	0.0007
Spinal surgery		189 (3.22)	4 (0.93)	0.0137
Orthopedic surgery		192 (3.27)	8 (1.86)	0.1512
Head and neck		226 (3.85)	13 (3.02)	0.4733
Other high-risk surgeries		59 (1.01)	15 (3.48)	<0.0001
Other low-risk surgeries		174 (2.97)	3 (0.70)	0.0108

Data are presented as mean ± SD, *n* (%). All *p* values are derived from Chi-squared tests

^*a*^*p* value derived from t-test

*BMI* body mass index, *ASAPS* American Society of Anesthesiologists Physical Status, *ACE* angiotensin-converting enzyme, *ARB* angiotensin receptor blocker, *CCB* calcium channel blocker, *BP* blood pressure, *eGFR* estimated glomerular filtration rate, *COPD* chronic obstructive pulmonary disease, *RBC* red blood cells, *FFP* fresh frozen plasma

### Univariable and multivariable logistic regression analyses (step 1)

The results of univariable logistic regression are shown for variables other than blood pressure (see Table 2S, Supplemental Digital Content 2). Table 2 shows the results of the univariable and multivariable logistic regression analyses. SBP <75 mmHg for more than 20 min was associated with postoperative AKI with an OR of 1.41 (95% CI 1.00–2.00) compared with SBP remaining above 75 mmHg. The results of the multivariable analysis of trend for an absolute threshold of 80 mmHg were statistically significant, suggesting that postoperative AKI risk increased as the duration of hypotension <80 mmHg increased. Also, SBP <70% of baseline for more than 20 min was associated with postoperative AKI with an OR of 1.58 (95% CI 1.03–2.52) compared with SBP remaining above 70% of baseline. The results of the multivariable analysis of trend for a relative threshold of 80% were statistically significant, suggesting that postoperative AKI risk increased as the duration of hypotension <80% increased. The results of the univariable and multivariable analyses showed similar tendencies regarding postoperative AKI. The covariates used in this multivariable logistic regression model were selected according to the stepwise method; the covariates and their estimators are shown for absolute thresholds (Table 3S, Supplemental Digital Content 3) and for relative thresholds (Table 4S, Supplemental Digital Content 4). Male sex; depression; use of beta blockers, diuretics, and/or CCBs; eGFR <45 ml min^−1^ 1.73m^−2^; longer anesthesia time; greater hemorrhage volume; and RBC transfusion were independently associated with postoperative AKI as indicated by multivariable logistic regression (*p*<0.05 in all cases). As for surgical procedures, laparoscopic major abdominal surgery, gynecological surgery, thoracic surgery, peripheral vascular surgery, neurosurgery, spinal surgery, orthopedic surgery, and other low-risk surgeries were associated with decreased risk of postoperative AKI compared with major abdominal surgery (*p*<0.05 in all cases).

**Table 2:** Univariable and multivariable associations between absolute or relative thresholds and acute kidney injury (*N*=6296)

	Duration	Total (*n*=6296)	AKI (*n*=431)	Unadjusted OR (95% CI)	Adjusted OR^a^ (95% CI)
**Absolute thresholds**					
<90 mmHg	0 min	222	14 (6.03%)	Ref.	Ref.
	1≤ <5 min	274	16 (5.84%)	0.92 (0.43 to 1.95)	0.78 (0.34 to 1.77)
	5≤ <20 min	997	55 (5.52%)	0.86 (0.48 to 1.63)	0.80 (0.42 to 1.63)
	20≤	4803	346 (7.21%)	1.14 (0.68 to 2.07)	0.80 (0.44 to 1.57)
	Trend			1.11 (0.97 to 1.30)	0.96 (0.82 to 1.14)
<85 mmHg	0 min	387	26 (6.72%)	Ref.	Ref.
	1≤ <5min	489	27 (5.52%)	0.81 (0.46 to 1.41)	0.69 (0.37 to 1.28)
	5≤ <20min	1593	72 (4.52%)	0.65 (0.41 to 1.05)	0.60 (0.37 to 1.03)
	20≤	3825	306 (8.00%)	1.20 (0.80 to 1.86)	0.86 (0.54 to 1.41)
	Trend			1.20 (1.06 to 1.36)	1.05 (0.92 to 1.21)
< 80 mmHg	0 min	668	39 (5.84%)	Ref.	Ref.
	1≤ <5 min	908	46 (5.07%)	0.86 (0.55 to 1.33)	0.79 (0.49 to 1.27)
	5≤ <20 min	2169	121 (5.58%)	0.95 (0.65 to 1.37)	0.87 (0.58 to 1.33)
	20≤	2551	225 (8.82%)	1.55 (1.09 to 2.21)	1.08 (0.74 to 1.63)
	Trend			1.24 (1.12 to 1.39)	1.08 (1.03 to 1.22)
<75 mmHg	0 min	1199	69 (5.75%)	Ref.	Ref.
	1≤ <5 min	1415	67 (4.73%)	0.81 (0.57 to 1.15)	0.79 (0.54 to 1.15)
	5≤ <20 min	2480	160 (6.45%)	1.12 (0.84 to 1.51)	1.04 (0.76 to 1.45)
	20≤	1202	135 (11.23%)	2.07 (1.54 to 2.82)	1.41 (1.00 to 2.00)
	Trend			1.32 (1.19 to 1.46)	1.16 (1.04 to 1.30)
< 70 mmHg	0 min	1980	114 (5.76%)	Ref.	Ref.
	1≤ <5 min	2071	118 (5.70%)	0.98 (0.75 to 1.28)	0.90 (0.68 to 1.20)
	5≤ <2 0min	1881	152 (8.08%)	1.43 (1.11 to 1.85)	1.09 (0.82 to 1.44)
	20≤	360	47 (12.91%)	2.45 (1.71 to 3.52)	1.60 (1.06 to 2.38)
	Trend			1.30 (1.17 to 1.44)	1.12 (1.00 to 1.26)
**Relative thresholds**					
< 85%	0 min	58	3 (4.92%)	Ref.	Ref.
	1≤ <5 min	113	7 (5.83%)	1.19 (0.32 to 5.71)	1.30 (0.27 to 9.55)
	5≤ <20 min	410	17 (3.98%)	0.80 (0.25 to 3.50)	0.98 (0.24 to 6.70)
	20≤	5284	404 (7.10%)	1.47 (0.54 to 6.07)	1.72 (0.48 to 11.14)
	Trend			1.36 (1.06 to 1.83)	1.32 (0.98 to 1.81)
<80%	0 min	133	7 (5.26%)	Ref.	Ref.
	1≤ <5 min	247	9 (3.64%)	0.68 (0.24 to 1.94)	0.68 (0.26 to 2.21)
	5≤ <20 min	664	38 (5.72%)	1.09 (0.50 to 2.72)	1.32 (0.54 to 3.74)
	20≤	5252	377 (7.18%)	1.39 (0.69 to 3.30)	1.58 (0.70 to 4.31)
	Trend			1.28 (1.06 to 1.56)	1.29 (1.05 to 1.59)
< 75%	0 min	278	13 (4.68%)	Ref.	Ref.
	1≤ <5 min	446	20 (4.48%)	0.95 (0.47 to 2.00)	0.94 (0.44 to 2.11)
	5≤ <20 min	1025	63 (6.15%)	1.33 (0.74 to 2.56)	1.44 (0.75 to 2.95)
	20≤	4547	335 (7.37%)	1.62 (0.95 to 3.00)	1.74 (0.96 to 3.45)
	Trend			1.24 (1.08 to 1.43)	1.25 (1.08 to 1.47)
<70%	0 min	532	27 (5.08%)	Ref.	Ref.
	1≤ <5 min	713	44 (6.17%)	1.23 (0.75 to 2.03)	1.14 (0.68 to 1.97)
	5≤ <20 min	1448	78 (5.39%)	1.06 (0.68 to 1.69)	1.09 (0.68 to 1.81)
	20≤	3603	282 (7.83)	1.58 (1.07 to 2.43)	1.58 (1.03 to 2.52)
	Trend			1.61 (0.73 to 3.21)	1.19 (1.06 to 1.35)
<65%	0 min	988	57 (5.77%)	Ref.	Ref.
	1≤ <5 min	1091	63 (5.77%)	1.00 (0.69 to 1.45)	0.99 (0.66 to 1.47)
	5≤ <20 min	1684	101 (6.00%)	1.04 (0.74 to 1.46)	1.08 (0.76 to 1.56)
	20≤	2533	210 (8.29%)	1.47 (1.09 to 2.01)	1.49 (1.07 to 2.09)
	Trend			1.16 (1.06 to 1.28)	1.16 (1.05 to 1.29)

^*a*^Adjusted for sex, age, ASAPS, beta blockers, CCBs, diuretics, baseline eGFR, hypertension, coronary artery disease, diabetes mellitus, cerebrovascular disease, mixed obstructive and restrictive disease, respiratory failure, hyperthyroidism, depression, ileus, anesthesia time, hemorrhage, crystalloid administration, RBC transfusion, urine as a categorical variable and surgical procedure type

*AKI* acute kidney injury, *OR* odds ratio, *ASAPS* American Society of Anesthesiologists Physical Status, *CCB* calcium channel blocker, *eGFR* estimated glomerular filtration rate, *RBC* red blood cells

### Examining whether the effects of crystalloid administration differ according to baseline renal function (step 2)

**Fig. 2:**
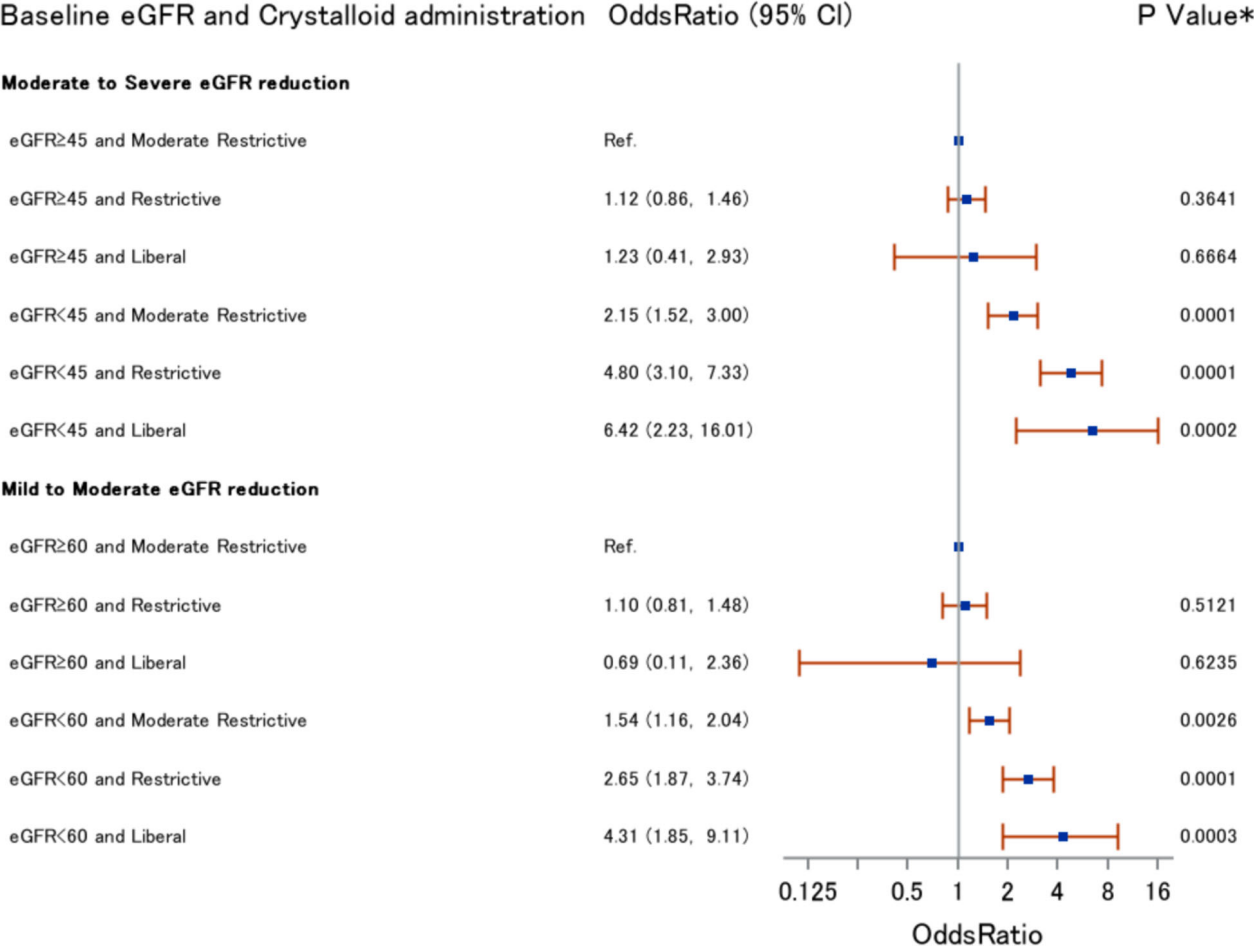
Different effects of crystalloid administration based on baseline renal function level (*N*=6296). “Moderately restrictive” refers to a crystalloid administration volume of >7 ml kg^−1^ h^−1^, ≤ 24 ml kg^−1^ h^−1^; “Liberal” refers to a crystalloid administration volume of >24 ml kg^−1^ h^−1^, and “Restrictive” refers to a crystalloid administration volume of ≤7 ml kg^−1^ h^−1^. eGFR, estimated glomerular filtration rate

We further examined the interactions between crystalloid administration and baseline renal function. We added an interaction term in the model used in step 1; otherwise, the covariates included in this model were the same as in step 1. In this model, we used an absolute blood pressure threshold of 80 mmHg. The following significant results were obtained. Postoperative AKI was more likely in patients with eGFR <45 ml min^−1^ 1.73m^−2^ and restrictive crystalloid administration than in patients with eGFR ≥45 ml min^−1^ 1.73m^−2^ and with moderately restrictive crystalloid administration, with an OR of 4.80 (95% CI 3.10–7.33). Postoperative AKI was more likely in patients with eGFR <45 ml min^−1^ 1.73m^−2^ and liberal crystalloid administration than in patients with eGFR ≥45 ml min^−1^ 1.73m^−2^ and with moderately restrictive crystalloid administration, with an OR of 6.42 (95% CI 2.23–16.01) (Fig. 2).

### Sensitivity analysis

The results of the univariable and multivariable logistic regression models in the sensitivity analysis using MAP instead of SBP are shown in Table 5S (Supplemental Digital Content 5). The trend analysis for MAP thresholds <55 mmHg indicated statistical significance with an OR of 1.16 (95% CI 1.02–1.33), indicating that postoperative AKI risk increased as the duration of hypotension <55 mmHg increased.

## Discussion

This study demonstrates that even patients undergoing elective noncardiac surgeries can be at increased risk of postoperative AKI if they are exposed to unintentional hypotension, especially when SBP falls below an absolute value of 80 mmHg or a relative value of 80% of baseline. We also found that both restrictive and liberal crystalloid administration were associated with postoperative AKI and that the effects of restrictive and liberal crystalloid administration differed significantly according to baseline renal function.

Our results are broadly consistent with previous studies in this field [6, 8, 11]. SBP below an absolute threshold of 80 mmHg or a relative threshold of 80% of baseline was associated with AKI. The absolute SBP threshold of 80 mmHg used in the present study is slightly lower than those used in some previous studies, but this can be partially explained by the fact that we included elective surgeries only. Another study that included elective surgeries only proposed a MAP threshold of 55–60 mmHg, which is consistent with our results [5]. One of the most important factors contributing to the diversity of results in previous studies may be differences among these studies in the degree of residual confounding, such as whether the studies adjusted for patient background factors and surgical procedures.

Both restrictive and liberal crystalloid administration have previously been reported to weaken renal function and to increase pulmonary complications and mortality. It is widely known that an appropriate quantity of crystalloid administration is essential to maintain renal perfusion. Yet there is still ongoing discussion as to how much crystalloid administration is restrictive and how much is liberal. While one recent large retrospective study showed a U-shaped association between the volume of crystalloid administration and postoperative 30-day mortality and morbidity including AKI [23], which is consistent with our study, some prospective studies have shown no difference between the effects of restrictive and moderately restrictive crystalloid administration on postoperative AKI [22, 24]. The main cause of these discrepancies could be differences in surgical procedures. In our study, the ORs of postoperative AKI following laparoscopic and thoracic surgery were lower than those following major abdominal surgery. The minimum required quantity of intraoperative crystalloid administration may differ for different surgical approaches.

Our analysis in step 2 suggests that the effect of crystalloid administration differs significantly according to baseline renal function. Restrictive and liberal crystalloid administration lead to markedly worse effects on patients with impaired renal function than on those without. Patients with eGFR <45 ml min^−1^ 1.73m^−2^ who were given restrictive or liberal crystalloid administration were 2.23 (4.80/2.15) or 2.98 (6.42/2.15) times as likely, respectively, to develop postoperative AKI compared with those with eGFR ≥45 ml min^−1^ 1.73m^−2^ who were treated with moderately restrictive crystalloid administration. Similar results were seen in patients with eGFR <60 ml min^−1^ 1.73m^−2^ and with eGFR <30 ml min^−1^ 1.73m^−2^ (results not shown). These differences were not only statistically but also clinically significant. We presume that the kidney becomes more susceptible to inappropriate crystalloid administration as the eGFR decreases. As far as we know, this is the first study to show that the effect of crystalloid administration differs significantly according to baseline renal function.

The effects of age and 0.9% saline were significant in univariable logistic regression but not significant in multivariable logistic regression. These variables were omitted from the final logistic regression model according to the stepwise method.

Our study has certain strengths. First, we excluded patients who had undergone emergency surgery because emergency surgery was a possible cause of reverse causality. Patients whose pre-operative conditions were severe enough to lead to postoperative AKI are more likely to experience low intraoperative blood pressure due to factors other than IOH such as sepsis. Septic patients are particularly susceptible to IOH, and a relatively high blood pressure should be maintained. Second, we adjusted for intraoperative crystalloid administration as a confounding factor although previous retrospective studies investigating blood pressure thresholds did not [6, 8, 9, 25].

Our study also has some weaknesses and limitations. First, it is subject to the inherent limitations associated with its single-center, retrospective, and observational design, such as limited generalizability, inability to prove causality, and unmeasured confounding factors. In particular, we did not have data on history of malignancy, which is a possible cause of postoperative AKI. We believe, however, that adjusting for surgical procedure type partially eliminates confounding by malignancy. Second, we used SBP as the main target of the analysis while using MAP for the sensitivity analysis. MAP is the most appropriate means of assessing tissue perfusion, but it is not always available in clinical practice, whereas SBP is almost always available. In fact, the Terumo ES-H55 electronic sphygmomanometer displays SBP but not MAP. Third, removing iatrogenic blood pressure fluctuations as artifacts may have caused us to underestimate the effect of IOH. However, there is no universal protocol for the removal of artifacts, and we excluded blood pressure data that were unlikely to occur in real patients. Fourth, we linearly interpolated non-invasive measurements into 1-min interval data. This may have caused us to overlook some short periods of IOH, but, given that abrupt changes of blood pressure are most common during induction of general anesthesia, which is normally a brief process, we believe it is unlikely that short-term measurements would noticeably change the effects of IOH. Fifth, we included both invasive and non-invasive blood pressure measurements. There is sometimes a gap between these measurements, particularly in critically ill patients whose systemic vascular resistance is abnormally high or low. However, our inclusion of elective surgeries only should minimize this blood pressure gap. Sixth, the minimum duration of hypotension that can trigger harm is unclear from this study. However, a graded relationship between the duration of hypotension and postoperative AKI has previously been assumed and is statistically supported by our study. Seventh, we used only creatinine instead of urine output to diagnose postoperative AKI due to the retrospective study design. This may have caused us to underestimate the incidence of postoperative AKI. Eighth, the appropriate volume of intraoperative crystalloid administration is not clear from this study. It should be determined for each case in light of the surgical procedure type and coexisting medical conditions such as baseline renal function. Ninth, appropriate organ perfusion was accomplished by optimizing intraoperative blood pressure, crystalloid administration, and catecholamine use, which are not available from this study. Despite these limitations, this study provides important evidence that anesthesiologists should maintain an absolute SBP >80 mmHg and avoid either restrictive or liberal crystalloid administration in patients with decreased eGFR. Intraoperative blood pressure and crystalloid administration are the major modifiable factors that must be optimized to prevent postoperative AKI. Future randomized trials should be conducted to determine the appropriate volume of intraoperative crystalloid administration based on each patient’s renal function and surgical procedure type.

## Conclusions

The results of our study strongly suggest that even patients undergoing elective surgery are at increased risk of postoperative AKI after being exposed to SBP <80 mmHg. Furthermore, the effects of restrictive and liberal crystalloid administration differed significantly according to baseline renal function. Our results highlight the importance of maintaining SBP >80 mmHg while administering an appropriate volume of crystalloid to prevent postoperative AKI.

## Supplementary Information

**Additional file 1: Supplemental Digital Content**. **Supplemental Digital Content 1, Table 1S**. The types of surgical procedures included in the “other high-risk surgeries” and “other low-risk surgeries” categories. **Supplemental Digital Content 2, Table 2S**. Univariable logistic regression analysis for acute kidney injury (N=6296). **Supplemental Digital Content 3**, **Table 3S**. All variables included in the multivariable logistic regression model for systolic blood pressure as a trend (absolute thresholds, N=6296). **Supplemental Digital Content 4, Table 4S**. All variables included in the multivariable logistic regression model for systolic blood pressure as a trend (relative thresholds, N=6296). **Supplemental Digital Content 5, Table 5S**. Univariable and multivariable associations between MAP absolute thresholds and acute kidney injury (N=6296).

## Data Availability

The datasets used and/or analyzed in the current study are available from the corresponding author on reasonable request.

## Abbreviations

AKI: Acute kidney injury
IOH: Intraoperative hypotension
eGFR: Estimated glomerular filtration rate
OR: Odds ratio
CI: Confidence interval
CKD: Chronic kidney disease
RCT: Randomized controlled trial
ASAPS: American Society of Anesthesiologists Physical Status
SBP: Systolic blood pressure
DBP: Diastolic blood pressure
BMI: Body mass index
ACE: Angiotensin-converting enzyme
ARBs: Angiotensin receptor blockers
CCBs: Calcium channel blockers
RBC: Red blood cells
FFP: Fresh frozen plasma
SD: Standardized deviation
MAP: Mean arterial pressure
