# Supplementary material for: Analysis of intraoperative modifiable factors to prevent acute kidney injury after elective noncardiac surgery: intraoperative hypotension and crystalloid administration related to acute kidney injury
Source: JA Clin Rep. 2021 Mar 24;7:27. doi: 10.1186/s40981-021-00429-9 (PMC7991025; doi:10.1186/s40981-021-00429-9)
Supplement: Supplementary file 1 — Additional file 1: Supplemental Digital Content. Supplemental Digital Content 1, Table 1S. The types of surgical procedures included in the “other high-risk surgeries” and “other low-risk surgeries” categories. Supplemental Digital Content 2, Table 2S. Univariable logistic regression analysis for acute kidney injury (N=6296). Supplemental Digital Content 3, Table 3S. All variables included in the multivariable logistic regression model for systolic blood pressure as a trend (absolute thresholds, N=6296). Supplemental Digital Content 4, Table 4S. All variables included in the multivariable logistic regression model for systolic blood pressure as a trend (relative thresholds, N=6296). Supplemental Digital Content 5, Table 5S. Univariable and multivariable associations between MAP absolute thresholds and acute kidney injury (N=6296). [file 40981_2021_429_MOESM1_ESM.docx]

| **Other high risk surgeries** | **Other low risk surgeries** |
| --- | --- |
| Abscess drainage, Debridement, Hemipelvectomy, Total pelvic exenteration, Tracheostomy, Transplantation of greater omentum | Arterial flap, Axillary lymph node biopsy, Axillary lymph node dissection, Bone marrow aspiration, Bone tumor biopsy, Dilational tracheostomy, Esophagogastroduodenal scope, Exploratory incision, Fistulectomy, Free skin graft, Full thickness skin graft, Inguinal hernia repair, Inguinal lymph node biopsy, Inguinal lymph node excision, Musculocutaneous flap, Open inguinal hernia repair, Other biopsy, Removal of foreign body by incision, Removal of pharyngeal foreign body, Removal of tracheal foreign body, Repair of umbilical hernia, Resection of soft tissue tumor, Resection of subcutaneous tumor, Skin biopsy, Skin graft, Skin tumor excision, Soft tissue tumor biopsy, Split thickness skin graft, Stripping of varicose vein, Stump plasty of extremities, Ventral hernia repair |

**Supplemental Digital Content 1, Table 1S. Each surgical procedure included in other high risk surgeries and other low risk surgeries.**

| **Supplemental Digital Content 2, Table 2S. Univariable logistic regression analysis for acute kidney injury (N=6296).** | | | | |
| --- | --- | --- | --- | --- |
| Variables |  | OR (95% CI) | P value | |
| **Baseline patients' characteristics** | | | | |
| Sex (female) |  | 0.57 (0.46 to 0.72) | <0.0001 | |
| Age, years | 50≤ <65 | Ref. |  | |
|  | 65≤ | 1.32 (1.04 to 1.68) | 0.0233 | |
| BMI, kg m^-2^ | <25 | Ref. |  | |
|  | 25≤ | 1.09 (0.88 to 1.35) | 0.4136 | |
| Body weight |  |  |  | |
| ASAPS | 2 | Ref. |  | |
|  | 3 | 1.72 (1.35 to 2.18) | <0.0001 | |
|  | 4 | 2.59 (1.05 to 5.47) | 0.0208 | |
| Cardiac medication histories |  |  |  | |
|  | ACE inhibitors | 1.87 (1.36 to 2.52) | <0.0001 | |
|  | ARBs | 1.34 (1.08 to 1.64) | <0.0062 | |
|  | Beta blockers | 2.07 (1.65 to 2.59) | <0.0001 | |
|  | CCBs | 1.63 (1.34 to 1.98) | <0.0001 | |
|  | Diuretics | 2.28 (1.73 to 2.97) | <0.0001 | |
| Baseline systolic BP, mmHg |  | 1.00 (0.99 to 1.00) | 0.1923 | |
| Baseline eGFR, ml min^-1^ 1.73m^-2^ | 45≤ | Ref. |  | |
|  | 15≤ <45 | 3.09 (2.45 to 3.88) | <0.0001 | |
| **Coexisting medical conditions** | | | | |
| Congestive heart failure |  | 1.48 (1.02 to 2.10) | 0.0335 | |
| Hypertension |  | 1.77 (1.44 to 2.19) | <0.0001 | |
| Coronary artery disease |  | 1.46 (1.09 to 1.92) | 0.0091 | |
| Valvular heart disease |  | 1.47 (1.04 to 2.03) | 0.0235 | |
| Diabetes mellitus |  | 1.34 (1.08 to 1.66) | <0.0074 | |
| Dyslipidemia |  | 1.07 (0.86 to 1.33) | 0.5210 | |
| Cerebrovascular disease |  | 1.57 (1.14 to 2.11) | 0.0038 | |
| Spinal cord injury |  | 2.27 (0.12 to 13.34) | | 0.4478 |
| COPD |  | 0.79 (0.59 to 1.05) | 0.1146 | |
| Interstitial pneumonia |  | 0.96 (0.63 to 1.40) | 0.8305 | |
| Mixed obstructive and restrictive lung disease | | 1.68 (0.84 to 3.03) | 0.1084 | |
| Respiratory failure |  | 0.60 (0.35 to 1.00) | 0.0501 | |
| Cirrhosis |  | 2.87 (1.41 to 5.33) | 0.0016 | |
| Coagulopathy |  | 1.47 (0.85 to 2.38) | 0.1362 | |
| Hyperthyroidism |  | 1.20 (0.58 to 2.19) | 0.5875 | |
| Neuromuscular disease |  | 0.42 (0.02 to 1.98) | 0.3987 | |
| Parkinson's disease |  | 2.28 (0.53 to 6.76) | 0.1881 | |
| Depression |  | 2.77 (1.36 to 5.12) | 0.0023 | |
| Schizophrenia |  | 0.72 (0.04 to 3.46) | 0.7451 | |
| Dementia |  | 0.66 (0.16 to 1.78) | 0.4791 | |
| Ileus |  | 2.74 (0.63 to 8.32) | 0.1121 | |
| **Intraoperative factors** | | | | |
| Anesthesia time, min | 0< ≤278 | Ref. |  | |
|  | 278< ≤372 | 1.14 (0.76 to 1.71) | 0.5299 | |
|  | 278< ≤501 | 2.71 (1.93 to 3.86) | <0.0001 | |
|  | 501< | 4.76 (3.46 to 6.68) | <0.0001 | |
| Hemorrhage, ml | 0 | Ref. |  | |
|  | 0< ≤100 | 0.96 (0.61 to 1.49) | 0.8386 | |
|  | 100< ≤330 | 2.04 (1.43 to 2.91) | <0.0001 | |
|  | 330< | 5.81 (4.22 to 8.17) | <0.0001 | |
| Crystalloid, ml kg^-1^ h^-1^ | 7≤ <24 | Ref. |  | |
|  | 24< | 0.98 (0.50 to 1.75) | 0.9580 | |
|  | 0≤ <7 | 1.34 (1.09 to 1.65) | 0.0050 | |
| 0.9% saline |  | 1.57 (1.29 to 1.91) | <0.0001 | |
| Synthetic colloid |  | 2.16 (1.77 to 2.63) | <0.0001 | |
| Transfusion (%) | RBC | 4.03 (3.18 to 5.07) | <0.0001 | |
|  | FFP | 4.53 (3.14 to 6.42) | <0.0001 | |
| Urine, ml kg^-1^ h^-1^ | 0.5≤ | Ref. |  | |
|  | 0≤ <0.5 | 1.20 (0.95 to 1.49) | 0.1059 | |
| Combined epidural anesthesia |  | 1.23 (1.01 to 1.50) | 0.0398 | |
| Use of arterial catheter |  | 1.89 (1.46 to 2.48) | <0.0001 | |
| **Surgical procedures** |  |  |  | |
| Major abdominal | | Ref. |  | |
| Laparoscopic major abdominal | | 0.27 (0.19 to 0.40) | <0.0001 | |
| Gynecological | | 0.24 (0.09 to 0.52) | 0.0010 | |
| Thoracic | | 0.17 (0.12 to 0.24) | <0.0001 | |
| Major vascular | | 1.01 (0.77 to 1.31) | 0.9255 | |
| Peripheral vascular | | 0.38 (0.20 to 0.68) | 0.0021 | |
| Neurosurgery | | 0.15 (0.07 to 0.29) | <0.0001 | |
| Spinal surgery | | 0.14 (0.04 to 0.33) | 0.0001 | |
| Orthopedic surgery | | 0.27 (0.12 to 0.53) | 0.0005 | |
| Head and Neck | | 0.38 (0.20 to 0.65) | 0.0011 | |
| Other high risk surgeries | | 1.69 (0.90 to 2.96) | 0.0797 | |
| Other low risk sugeries | | 0.11 (0.02 to 0.30) | 0.0002 | |
| OR = odds ratio; BMI = body mass index; ASAPS = American Society of Anesthesiologists Physical Status; ACE = angiotensin to converting enzyme; ARB = angiotensin receptor blocker; CCB = calcium channel blocker; BP = blood pressure; eGFR = estimated glomerular filtration rate; COPD = chronic obstructive pulmonary disease; RBC = red blood cells; FFP = fresh frozen plasma. | | | | |

| **Supplemental Digital Content 3, Table 3S. All variables included in the multivariable logistic regression model for systolic blood pressure as a trend (absolute thresholds, N=6296).** | | | | | | | | |  |
| --- | --- | --- | --- | --- | --- | --- | --- | --- | --- |
| Variables | | | | Absolute blood pressure thresholds: OR (95% CI) | | | | | |
|  |  |  |  | 70mmHg | 75mmHg | 80mmHg | 85mmHg | 90mmHg | |
| Blood pressure (Trend) | | |  | 1.12 (1.00 to 1.26) | 1.16 (1.04 to 1.30) | 1.08 (1.03 to 1.22) | 1.05 (0.92 to 1.21) | 0.96 (0.82 to 1.14) | |
| Female | |  | | 0.74 (0.57 to 0.95) | 0.73 (0.56 to 0.95) | 0.74 (0.57 to 0.95) | 0.74 (0.57 to 0.95) | 0.74 (0.57 to 0.96) | |
| Age, years | 50≤ <65 | | | Ref. | Ref. | Ref. | Ref. | Ref. | |
|  | 65≤ | | | 0.90 (0.69 to 1.17) | 0.89 (0.68 to 1.17) | 0.90 (0.69 to 1.18) | 0.91 (0.70 to 1.19) | 0.91 (0.70 to 1.20) | |
| ASAPS | 2 | | | Ref. | Ref. | Ref. | Ref. | Ref. | |
|  | 3 | | | 1.45 (1.10 to 1.90) | 1.46 (1.10 to 1.91) | 1.45 (1.10 to 1.90) | 1.45 (1.10 to 1.90) | 1.44 (1.09 to 1.88) | |
|  | 4 | | | 2.51 (0.94 to 5.92) | 2.57 (0.96 to 6.05) | 2.47 (0.92 to 5.83) | 2.45 (0.92 to 5.77) | 2.38 (0.89 to 5.62) | |
| Cardiac medication histories |  | | |  |  |  |  |  | |
|  | Beta blockers | | | 1.42 (1.08 to 1.86) | 1.42 (1.08 to 1.86) | 1.42 (1.08 to 1.85) | 1.42 (1.08 to 1.85) | 1.41 (1.08 to 1.85) | |
|  | CCBs | | | 1.31 (1.02 to 1.68) | 1.32 (1.03 to 1.69) | 1.30 (1.02 to 1.67) | 1.30 (1.02 to 1.67) | 1.29 (1.01 to 1.65) | |
|  | Diuretics | | | 1.48 (1.07 to 2.01) | 1.49 (1.08 to 2.03) | 1.48 (1.08 to 2.02) | 1.48 (1.08 to 2.01) | 1.47 (1.07 to 2.00) | |
| Baseline eGFR, ml min^-1^ 1.73m^-2^ | 45≤ | | | Ref. | Ref. | Ref. | Ref. | Ref. | |
|  | 15≤ <45 | | | 2.74 (2.08 to 3.60) | 2.76 (2.09 to 3.63) | 2.76 (2.09 to 3.63) | 2.75 (2.09 to 3.62) | 2.73 (2.07 to 3.58) | |
| Hypertension | | |  | 1.22 (0.93 to 1.60) | 1.22 (0.93 to 1.59) | 1.22 (0.93 to 1.59) | 1.21 (0.93 to 1.59) | 1.21 (0.93 to 1.59) | |
| Coronary artery disease | | |  | 0.78 (0.55 to 1.08) | 0.79 (0.56 to 1.09) | 0.78 (0.55 to 1.08) | 0.77 (0.55 to 1.07) | 0.75 (0.54 to 1.05) | |
| Diabetes mellitus | | |  | 1.19 (0.94 to 1.51) | 1.19 (0.94 to 1.51) | 1.19 (0.93 to 1.50) | 1.19 (0.94 to 1.50) | 1.18 (0.93 to 1.49) | |
| Cerebrovascular disease | | |  | 1.28 (0.90 to 1.80) | 1.30 (0.91 to 1.83) | 1.29 (0.90 to 1.81) | 1.28 (0.89 to 1.79) | 1.27 (0.89 to 1.78) | |
| Mixed obstructive and restrictive lung disease | | | | 2.00 (0.94 to 3.91) | 1.99 (0.93 to 3.88) | 2.02 (0.95 to 3.93) | 2.04 (0.96 to 3.96) | 2.05 (0.97 to 3.98) | |
| Respiratory failure | | |  | 0.63 (0.35 to 1.07) | 0.64 (0.36 to 1.08) | 0.63 (0.35 to 1.07) | 0.63 (0.35 to 1.05) | 0.63 (0.35 to 1.05) | |
| Hyperthyroidism | | |  | 1.59 (0.72 to 3.18) | 1.57 (0.71 to 3.15) | 1.59 (0.72 to 3.18) | 1.60 (0.72 to 3.19) | 1.61 (0.73 to 3.20) | |
| Depression | | |  | 4.09 (1.86 to 8.30) | 4.12 (1.88 to 8.37) | 4.11 (1.87 to 8.33) | 4.13 (1.88 to 8.38) | 4.16 (1.89 to 8.45) | |
| Ileus | |  | | 2.65 (0.58 to 8.90) | 2.70 (0.59 to 9.07) | 2.66 (0.57 to 8.95) | 2.64 (0.57 to 8.90) | 2.67 (0.58 to 8.97) | |
| Anesthesia time, min | | 0< ≤278 | | Ref. | Ref. | Ref. | Ref. | Ref. | |
|  | | 278< ≤372 | | 1.01 (0.63 to 1.64) | 1.00 (0.62 to 1.62) | 1.01 (0.63 to 1.64) | 1.02 (0.63 to 1.66) | 1.04 (0.64 to 1.69) | |
|  | | 278< ≤501 | | 1.51 (0.92 to 2.49) | 1.47 (0.90 to 2.43) | 1.50 (0.92 to 2.48) | 1.52 (0.93 to 2.51) | 1.57 (0.96 to 2.60) | |
|  | | 501< | | 1.93 (1.15 to 3.27) | 1.88 (1.12 to 3.19) | 1.93 (1.15 to 3.28) | 1.97 (1.17 to 3.34) | 2.05 (1.22 to 3.48) | |
| Hemorrhage, mL | | 0 | | Ref. | Ref. | Ref. | Ref. | Ref. | |
|  | | 0< ≤100 | | 0.90 (0.55 to 1.46) | 0.90 (0.55 to 1.46) | 0.90 (0.55 to 1.46) | 0.90 (0.55 to 1.46) | 0.90 (0.55 to 1.45) | |
|  | | 100< ≤330 | | 1.60 (1.01 to 2.57) | 1.60 (1.01 to 2.57) | 1.59 (1.00 to 2.56) | 1.59 (1.00 to 2.55) | 1.58 (1.00 to 2.54) | |
|  | | 330< | | 2.85 (1.75 to 4.73) | 2.82 (1.73 to 4.68) | 2.85 (1.75 to 4.72) | 2.86 (1.75 to 4.73) | 2.86 (1.75 to 4.74) | |
| Crystalloid, ml kg^-1^ h^-1^ | | 7≤ <24 | | Ref. | Ref. | Ref. | Ref. | Ref. | |
|  | | 24< | | 1.69 (0.81 to 3.24) | 1.69 (0.81 to 3.23) | 1.71 (0.82 to 3.27) | 1.72 (0.82 to 3.28) | 1.73 (0.83 to 3.30) | |
|  | | 0≤ <7 | | 1.30 (1.03 to 1.64) | 1.30 (1.03 to 1.64) | 1.30 (1.03 to 1.64) | 1.30 (1.02 to 1.64) | 1.30 (1.02 to 1.64) | |
| Transfusion | | RBC | | 1.57 (1.18 to 2.08) | 1.57 (1.18 to 2.08) | 1.59 (1.20 to 2.11) | 1.60 (1.20 to 2.12) | 1.61 (1.21 to 2.13) | |
| Urine, ml kg^-1^ h^-1^ | | 0.5≤ | | Ref. | Ref. | Ref. | Ref. | Ref. | |
|  | | 0≤ <0.5 | | 1.76 (1.36 to 2.27) | 1.75 (1.35 to 2.26) | 1.75 (1.35 to 2.26) | 1.76 (1.36 to 2.27) | 1.77 (1.37 to 2.29) | |
| Surgical procedures | | Major abdominal | | Ref. | Ref. | Ref. | Ref. | Ref. | |
|  | | Laparoscopic major abdominal | | 0.56 (0.36 to 0.86) | 0.56 (0.36 to 0.86) | 0.55 (0.35 to 0.85) | 0.55 (0.35 to 0.84) | 0.54 (0.34 to 0.83) | |
|  | | Gynecological | | 0.41 (0.15 to 0.92) | 0.41 (0.15 to 0.93) | 0.41 (0.15 to 0.92) | 0.40 (0.15 to 0.92) | 0.40 (0.15 to 0.91) | |
|  | | Thoracic | | 0.35 (0.23 to 0.51) | 0.35 (0.23 to 0.51) | 0.35 (0.23 to 0.51) | 0.35 (0.23 to 0.51) | 0.35 (0.23 to 0.51) | |
|  | | Major vascular | | 0.77 (0.56 to 1.06) | 0.77 (0.56 to 1.06) | 0.77 (0.56 to 1.06) | 0.77 (0.56 to 1.06) | 0.77 (0.56 to 1.06) | |
|  | | Peripheral vascular | | 0.45 (0.22 to 0.84) | 0.45 (0.22 to 0.84) | 0.44 (0.22 to 0.83) | 0.44 (0.22 to 0.82) | 0.44 (0.22 to 0.81) | |
|  | | Neurosurgery | | 0.20 (0.09 to 0.41) | 0.21 (0.09 to 0.41) | 0.20 (0.09 to 0.41) | 0.20 (0.09 to 0.40) | 0.20 (0.08 to 0.39) | |
|  | | Spinal surgery | | 0.19 (0.05 to 0.48) | 0.20 (0.06 to 0.49) | 0.20 (0.06 to 0.50) | 0.20 (0.06 to 0.50) | 0.20 (0.06 to 0.49) | |
|  | | Orthopedic surgery | | 0.46 (0.20 to 0.95) | 0.46 (0.20 to 0.95) | 0.46 (0.20 to 0.95) | 0.46 (0.19 to 0.94) | 0.45 (0.19 to 0.93) | |
|  | | Head and Neck | | 0.71 (0.35 to 1.32) | 0.70 (0.35 to 1.32) | 0.70 (0.35 to 1.30) | 0.70 (0.35 to 1.30) | 0.70 (0.35 to 1.30) | |
|  | | Other high risk surgeries | | 1.76 (0.86 to 3.44) | 1.79 (0.87 to 3.49) | 1.76 (0.86 to 3.44) | 1.75 (0.85 to 3.41) | 1.69 (0.82 to 3.31) | |
|  | | Other low risk sugeries | | 0.20 (0.05 to 0.59) | 0.20 (0.05 to 0.59) | 0.20 (0.04 to 0.59) | 0.20 (0.05 to 0.59) | 0.21 (0.05 to 0.59) | |
| OR = odds ratio; ASAPS = American Society of Anesthesiologists Physical Status; CCB = calcium channel blocker; eGFR = estimated glomerular filtration rate; RBC = red blood cells. | | | | | | | | | |

| **Supplemental Digital Content 4, Table 4S. All variables included in the multivariable logistic regression model for systolic blood pressure as a trend (relative thresholds, N=6296).** | | | | | | | |
| --- | --- | --- | --- | --- | --- | --- | --- |
| Variables | | | Relative blood pressure thresholds: OR (95% CI) | | | | |
|  |  |  | 65% | 70% | 75% | 80% | 85% |
| Blood pressure (Trend) | |  | 1.16 (1.05 to 1.29) | 1.19 (1.06 to 1.35) | 1.25 (1.08 to 1.47) | 1.29 (1.05 to 1.59) | 1.32 (0.98 to 1.81) |
| Female | |  | 0.74 (0.57 to 0.95) | 0.74 (0.57 to 0.95) | 0.74 (0.57 to 0.95) | 0.74 (0.57 to 0.95) | 0.74 (0.57 to 0.95) |
| Age, years | 50≤ <65 | | Ref. | Ref. | Ref. | Ref. | Ref. |
|  | 65≤ | | 0.89 (0.69 to 1.17) | 0.90 (0.69 to 1.18) | 0.90 (0.69 to 1.18) | 0.91 (0.70 to 1.19) | 0.91 (0.70 to 1.20) |
| ASAPS | 2 | | Ref. | Ref. | Ref. | Ref. | Ref. |
|  | 3 | | 1.46 (1.11 to 1.92) | 1.47 (1.11 to 1.93) | 1.48 (1.12 to 1.94) | 1.47 (1.11 to 1.92) | 1.46 (1.10 to 1.91) |
|  | 4 | | 2.50 (0.93 to 5.89) | 2.54 (0.94 to 6.01) | 2.57 (0.96 to 6.07) | 2.61 (0.97 to 6.16) | 2.57 (0.96 to 6.06) |
| Cardiac medication histories | | |  |  |  |  |  |
|  | Beta blockers | | 1.43 (1.08 to 1.87) | 1.44 (1.09 to 1.88) | 1.44 (1.10 to 1.89) | 1.44 (1.09 to 1.89) | 1.43 (1.09 to 1.88) |
|  | CCBs | | 1.30 (1.02 to 1.67) | 1.29 (1.01 to 1.66) | 1.29 (1.01 to 1.65) | 1.29 (1.01 to 1.65) | 1.28 (1.00 to 1.64) |
|  | Diuretics | | 1.53 (1.11 to 2.09) | 1.52 (1.11 to 2.08) | 1.52 (1.11 to 2.08) | 1.53 (1.11 to 2.08) | 1.51 (1.10 to 2.06) |
| Baseline eGFR, ml min^-1^ 1.73m^-2^ | 45≤ | | Ref. | Ref. | Ref. | Ref. | Ref. |
|  | 15≤ <45 | | 2.75 (2.09 to 3.62) | 2.78 (2.10 to 3.65) | 2.75 (2.09 to 3.62) | 2.73 (2.07 to 3.59) | 2.73 (2.07 to 3.59) |
| Hypertension | |  | 1.20 (0.92 to 1.571) | 1.19 (0.91 to 1.56) | 1.20 (0.91 to 1.57) | 1.20 (0.92 to 1.58) | 1.21 (0.92 to 1.58) |
| Coronary artery diasease | |  | 0.79 (0.56 to 1.11) | 0.79 (0.56 to 1.10) | 0.79 (0.56 to 1.10) | 0.79 (0.56 to 1.09) | 0.78 (0.55 to 1.08) |
| Diabetes mellitus | |  | 1.20 (0.94 to 1.52) | 1.20 (0.94 to 1.51) | 1.20 (0.94 to 1.52) | 1.19 (0.94 to 1.51) | 1.19 (0.94 to 1.51) |
| Cerebrovascular disease | |  | 1.27 (0.88 to 1.78) | 1.28 (0.89 to 1.79) | 1.28 (0.90 to 1.80) | 1.28 (0.90 to 1.80) | 1.29 (0.90 to 1.81) |
| Mixed obstructive and restrictive lung disease | | | 2.02 (0.95 to 3.95) | 2.03 (0.96 to 3.95) | 2.05 (0.97 to 3.99) | 2.05 (0.96 to 3.99) | 2.04 (0.96 to 3.97) |
| Respiratory failure | |  | 0.64 (0.36 to 1.08) | 0.64 (0.35 to 1.07) | 0.63 (0.35 to 1.06) | 0.62 (0.35 to 1.05) | 0.62 (0.34 to 1.05) |
| Hyperthyroidism | |  | 1.58 (0.71 to 3.15) | 1.61 (0.73 to 3.21) | 1.62 (0.73 to 3.23) | 1.61 (0.73 to 3.21) | 1.58 (0.71 to 3.16) |
| Depression | |  | 4.08 (1.86 to 8.28) | 4.06 (1.85 to 8.23) | 4.11 (1.87 to 8.34) | 4.08 (1.85 to 8.29) | 4.11 (1.87 to 8.36) |
| Ileus | |  | 2.74 (0.60 to 9.12) | 2.75 (0.60 to 9.14) | 2.71 (0.59 to 9.05) | 2.63 (0.57 to 8.81) | 2.71 (0.59 to 9.07) |
| Anesthesia time, min | 0< ≤278 | | Ref. | Ref. | Ref. | Ref. | Ref. |
|  | 278< ≤372 | | 1.01 (0.62 to 1.63) | 1.01 (0.63 to 1.64) | 1.02 (0.63 to 1.65) | 1.03 (0.64 to 1.67) | 1.03 (0.64 to 1.67) |
|  | 278< ≤501 | | 1.49 (0.91 to 2.45) | 1.49 (0.91 to 2.45) | 1.48 (0.91 to 2.45) | 1.50 (0.92 to 2.47) | 1.52 (0.93 to 2.50) |
|  | 501< | | 1.91 (1.14 to 3.24) | 1.90 (1.14 to 3.23) | 1.90 (1.14 to 3.23) | 1.93 (1.15 to 3.27) | 1.96 (1.18 to 3.32) |
| Hemorrhage, ml | 0 | | Ref. | Ref. | Ref. | Ref. | Ref. |
|  | 0< ≤100 | | 0.90 (0.55 to 1.46) | 0.89 (0.55 to 1.45) | 0.89 (0.54 to 1.44) | 0.88 (0.54 to 1.44) | 0.89 (0.54 to 1.44) |
|  | 100< ≤330 | | 1.59 (1.00 to 2.56) | 1.59 (1.00 to 2.55) | 1.59 (1.00 to 2.55) | 1.59 (1.00 to 2.55) | 1.58 (1.00 to 2.54) |
|  | 330< | | 2.82 (1.73 to 4.68) | 2.82 (1.73 to 4.68) | 2.83 (1.73 to 4.69) | 2.84 (1.74 to 4.70) | 2.83 (1.74 to 4.70) |
| Crystalloid, ml kg^-1^ h^-1^ | 7≤ <24 | | Ref. | Ref. | Ref. | Ref. | Ref. |
|  | 24< | | 1.69 (0.81 to 3.24) | 1.69 (0.81 to 3.24) | 1.69 (0.81 to 3.24) | 1.71 (0.82 to 3.26) | 1.71 (0.82 to 3.26) |
|  | 0≤ <7 | | 1.30 (1.03 to 1.64) | 1.30 (1.03 to 1.65) | 1.30 (1.03 to 1.64) | 1.30 (1.02 to 1.64) | 1.30 (1.02 to 1.64) |
| Transfusion | RBC | | 1.60 (1.20 to 2.12) | 1.60 (1.20 to 2.12) | 1.61 (1.21 to 2.13) | 1.60 (1.20 to 2.12) | 1.61 (1.21 to 2.13) |
| Urine, ml kg^-1^ h^-1^ | 0.5≤ | | Ref. | Ref. | Ref. | Ref. | Ref. |
|  | 0≤ <0.5 | | 1.72 (1.32 to 2.22) | 1.73 (1.33 to 2.23) | 1.73 (1.34 to 2.24) | 1.75 (1.35 to 2.26) | 1.76 (1.35 to 2.27) |
| Surgical procedures | Major abdominal | | Ref. | Ref. | Ref. | Ref. | Ref. |
|  | Laparoscopic major abdominal | | 0.54 (0.35 to 0.83) | 0.54 (0.34 to 0.82) | 0.54 (0.34 to 0.82) | 0.54 (0.34 to 0.82) | 0.54 (0.35 to 0.83) |
|  | Gynecological | | 0.40 (0.15 to 0.91) | 0.39 (0.14 to 0.90) | 0.39 (0.14 to 0.88) | 0.39 (0.14 to 0.90) | 0.40 (0.14 to 0.90) |
|  | Thoracic | | 0.34 (0.23 to 0.50) | 0.34 (0.23 to 0.50) | 0.34 (0.23 to 0.50) | 0.34 (0.23 to 0.50) | 0.34 (0.23 to 0.50) |
|  | Major vascular | | 0.78 (0.56 to 1.07) | 0.77 (0.56 to 1.06) | 0.77 (0.56 to 1.05) | 0.76 (0.55 to 1.05) | 0.76 (0.55 to 1.05) |
|  | Peripheral vascular | | 0.42 (0.21 to 0.78) | 0.42 (0.20 to 0.78) | 0.41 (0.20 to 0.77) | 0.42 (0.21 to 0.78) | 0.42 (0.21 to 0.79) |
|  | Neurosurgery | | 0.19 (0.08 to 0.38) | 0.19 (0.08 to 0.38) | 0.19 (0.08 to 0.38) | 0.19 (0.08 to 0.38) | 0.19 (0.08 to 0.39) |
|  | Spinal surgery | | 0.19 (0.05 to 0.47) | 0.19 (0.05 to 0.47) | 0.19 (0.05 to 0.47) | 0.19 (0.05 to 0.48) | 0.19 (0.06 to 0.48) |
|  | Orthopedic surgery | | 0.45 (0.19 to 0.92) | 0.45 (0.19 to 0.92) | 0.45 (0.19 to 0.92) | 0.45 (0.19 to 0.93) | 0.46 (0.20 to 0.95) |
|  | Head and Neck | | 0.67 (0.33 to 1.25) | 0.66 (0.33 to 1.23) | 0.65 (0.32 to 1.22) | 0.66 (0.33 to 1.24) | 0.68 (0.34 to 1.27) |
|  | Other high risk surgeries | | 1.75 (0.85 to 3.42) | 1.73 (0.84 to 3.38) | 1.73 (0.84 to 3.38) | 1.75 (0.85 to 3.42) | 1.71 (0.83 to 3.34) |
|  | Other low risk sugeries | | 0.21 (0.05 to 0.60) | 0.20 (0.04 to 0.59) | 0.20 (0.04 to 0.58) | 0.20 (0.04 to 0.59) | 0.21 (0.05 to 0.59) |
| OR = odds ratio; ASAPS = American Society of Anesthesiologists Physical Status; CCB = calcium channel blocker; eGFR = estimated glomerular filtration rate; RBC = red blood cells. | | | | | | | |
|  | | | | | | | |
|  |  |  |  |  |  |  |  |

| \| **Supplemental Digital Content 5, Table 5S. Univariable and multivariable associations between MAP absolute thresholds and acute kidney injury (N=6296).** \| \| \| \| \| \| \| \| --- \| --- \| --- \| --- \| --- \| --- \| --- \| \|  \|  \| Duration \| Total (n=6296) \| AKI (n=431) \| Unadjusted OR (95% CI) \| Adjusted OR*^a^* (95% CI) \| \| Absolute thresholds \| \| \| \| \| \| \| \|  \| <65mmHg \| 0min \| 109 \| 3 (2.75%) \| Ref. \| Ref. \| \| 1≤ <5min \| 150 \| 8 (5.33%) \| 1.98 (0.56 to 9.25) \| 1.70 (0.45 to 8.24) \| \| 5≤ <20min \| 582 \| 24 (4.12%) \| 1.45 (0.49 to 6.21) \| 1.37 (0.45 to 6.02) \| \| 20≤ \| 5455 \| 7.26 (7.26%) \| 2.77 (1.03 to 11.30) \| 1.35 (0.48 to 5.66) \| \| Trend \| \| \| 1.45 (1.16 to 1.88) \| 0.99 (0.79 to 1.29) \| \| <60mmHg \| 0min \| 243 \| 7 (2.88%) \| Ref. \| Ref. \| \| 1≤ <5min \| 416 \| 19 (4.57%) \| 1.88 (0.78 to 5.22) \| 1.92 (0.76 to 5.51) \| \| 5≤ <20min \| 1235 \| 57 (4.62%) \| 1.90 (0.87 to 4.98) \| 1.80 (0.79 to 4.88) \| \| 20≤ \| 4402 \| 348 (7.91%) \| 3.38 (1.63 to 8.63) \| 1.79 (0.82 to 4.76) \| \| Trend \| \| \| 1.51 (1.28 to 1.79) \| 1.06 (0.89 to 1.27) \| \| <55mmHg \| 0min \| 560 \| 14 (2.50%) \| Ref. \| Ref. \| \| 1≤ <5min \| 941 \| 42 (4.46%) \| 1.96 (1.07 to 3.83) \| 1.68 (0.89 to 3.37) \| \| 5≤ <20min \| 2009 \| 111 (5.53%) \| 2.45 (1.42 to 4.61) \| 1.88 (1.05 to 3.62) \| \| 20≤ \| 2786 \| 264 (9.48%) \| 4.41 (2.61 to 8.17) \| 2.05 (1.16 to 3.92) \| \| Trend \| \| \| 1.60 (1.42 to 1.82) \| 1.16 (1.02 to 1.33) \| \| <50mmHg \| 0min \| 1154 \| 43 (3.73%) \| Ref. \| Ref. \| \| 1≤ <5min \| 1865 \| 100 (5.36%) \| 1.49 (1.04 to 2.18) \| 1.11 (0.75 to 1.67) \| \| 5≤ <20min \| 2199 \| 163 (7.41%) \| 2.12 (1.51 to 3.03) \| 1.30 (0.90 to 1.93) \| \| 20≤ \| 1078 \| 125 (11.60%) \| 3.51 (2.47 to 5.08) \| 1.45 (0.97 to 2.19) \| \| Trend \| \| \| 1.51 (1.36 to 1.68) \| 1.13 (1.01 to 1.28) \| \| <45mmHg \| 0min \| 2084 \| 93 (4.46%) \| Ref. \| Ref. \| \| 1≤ <5min \| 2583 \| 171 (6.59%) \| 1.53 (1.18 to 1.99) \| 1.03 (0.77 to 1.37) \| \| 5≤ <20min \| 1391 \| 133 (9.56%) \| 2.29 (1.74 to 3.02) \| 1.22 (0.89 to 1.66) \| \| 20≤ \| 228 \| 34 (14.91%) \| 3.79 (2.46 to 5.72) \| 1.49 (0.92 to 2.36) \| \| Trend \| \| \| 1.53 (1.37 to 1.71) \| 1.13 (0.99 to 1.29) \| \| *^a^*Adjusted for sex, age, ASAPS, beta blockers, CCB, diuretics, baseline eGFR, hypertension, coronary artery disease, diabetes mellitus, cerebrovascular disease, mixed obstructive and restrictive disease, respiratory failure, hyperthyroidism, depression, ileus, anesthesia time, hemorrhage, crystalloid administration, RBC transfusion, urine as categorical variable and surgical procedures. \| \| \| \| \| \| \| \| MAP = mean arterial pressure; ASAPS = American Socieity of Anesthesiologists Physical Status; CCB = calcium channel blocker; eGFR = estimated glomerular filtration rate; RBC = red blood cells. \| \| \| \| \| \| \| \| |
| --- | --- | --- | --- | --- | --- | --- | --- | --- | --- | --- | --- | --- | --- | --- | --- | --- | --- | --- | --- | --- | --- | --- | --- | --- | --- | --- | --- | --- | --- | --- | --- | --- | --- | --- | --- | --- | --- | --- | --- | --- | --- | --- | --- | --- | --- | --- | --- | --- | --- | --- | --- | --- | --- | --- | --- | --- | --- | --- | --- | --- | --- | --- | --- | --- | --- | --- | --- | --- | --- | --- | --- | --- | --- | --- | --- | --- | --- | --- | --- | --- | --- | --- | --- | --- | --- | --- | --- | --- | --- | --- | --- | --- | --- | --- | --- | --- | --- | --- | --- | --- | --- | --- | --- | --- | --- | --- | --- | --- | --- | --- | --- | --- | --- | --- | --- | --- | --- | --- | --- | --- | --- | --- | --- | --- | --- | --- | --- | --- | --- | --- | --- | --- | --- | --- | --- | --- | --- | --- | --- | --- | --- | --- | --- | --- | --- | --- | --- | --- | --- | --- | --- | --- | --- | --- | --- | --- | --- | --- | --- | --- | --- | --- | --- | --- | --- | --- |
